# The Good Life program for healthy brain aging is an active registry for clinical research recruitment

**DOI:** 10.1002/alz.093528

**Published:** 2025-01-09

**Authors:** David K Johnson

**Affiliations:** ^1^ UC Davis Alzheimer’s Disease Center, Walnut Creek, CA USA

## Abstract

**Background:**

*The Good Life* program for healthy brain aging (**TGL**) is an online program for positive lifestyle change that is made up of exercise, diet and social support classes. Since 2020, *The Good Life* has served thousands of older Californians. Currently, we host 17 classes weekly for our 750 active members (in both English and Spanish). Our exercise and cooking classes are our most watched content. Exercise classes attendance is 20‐70 participants per show (x11 weekly). Our x3/week cooking classes attendance is 20‐50 participants per show. We provide *The Good Life* freely and, in the process, engender goodwill ‐ a proven pathway to rebuilding trust and fostering reciprocity. *The Good Life* enjoys funding support from the State of California as public health program to support Black and Latinx healthy aging. In 2023 it expanded broadly across 13 California counties participating in the CDC’s Healthy Bain Initiative, providing TGL to all California residents. Three of the 13 counties are now creating local content tailor made to their specific ethno‐geographies.

**Method:**

TGL is implementing a 9‐step infrastructure building plan *to* (1) **Extend** this California program to scale nationally to serve large numbers of participants in NIA clinical research and training network centers; (2) **Engage** Black and Latinx American communities across the nation in a thoughtful, fun, and clinically meaningful health education campaign with messaging that will facilitate recruitment and enhance retention; (3) **Evaluate** the success of our precision engagement program using empirical metrics: (i) participant enrollment into clinical trials, (ii) time spent watching content per digital segment, (iii) number of clicks into subsequent public health messaging. These metrics will be correlated with participant data describing changes in attitudes re (a) Trust in Biomedical Research and (b) Stages of Research Volunteerism.

**Result:**

Since May 2023, TGL has recruited 260 Black Americans into 3 academic clinical research programs.

**Conclusion:**

TGL is a scalable internet‐based program with potential reach well beyond its large existing membership.

